# High-G MEMS Accelerometer Calibration Denoising Method Based on EMD and Time-Frequency Peak Filtering

**DOI:** 10.3390/mi14050970

**Published:** 2023-04-28

**Authors:** Chenguang Wang, Yuchen Cui, Yang Liu, Ke Li, Chong Shen

**Affiliations:** 1 School of Information and Communication Engineering, North University of China, Taiyuan 030051, China; wangchenguang@nuc.edu.cn; 2 Science and Technology on Electronic Test & Measurement Laboratory, North University of China, Taiyuan 030051, China; 2006040621@st.nuc.edu.cn; 3 School of Instrument and Electronics, North University of China, Taiyuan 030051, China; 4 Shanxi North Machine-Building Co., Ltd., Taiyuan 030051, China; m18734913028@163.com

**Keywords:** MEMS accelerometer, empirical mode decomposition, time-frequency peak filtering, high-g calibration

## Abstract

In order to remove noise generated during the accelerometer calibration process, an accelerometer denoising method based on empirical mode decomposition (EMD) and time-frequency peak filtering (TFPF) is proposed in this paper. Firstly, a new design of the accelerometer structure is introduced and analyzed by finite element analysis software. Then, an algorithm combining EMD and TFPF is proposed for the first time to deal with the noise of the accelerometer calibration process. Specific steps taken are to remove the intrinsic mode function (IMF) component of the high frequency band after the EMD decomposition, and then to use the TFPF algorithm to process the IMF component of the medium frequency band; meanwhile, the IMF component of the low frequency band is reserved, and finally the signal is reconstructed. The reconstruction results show that the algorithm can effectively suppress the random noise generated during the calibration process. The results of spectrum analysis show that EMD + TFPF can effectively protect the characteristics of the original signal and that the error can be controlled within 0.5%. Finally, Allan variance is used to analyze the results of the three methods to verify the filtering effect. The results show that the filtering effect of EMD + TFPF is the most obvious, being 97.4% higher than the original data.

## 1. Introduction

Inertial technology is a cross-integrated technology involving inertial navigation [1] and guidance [2], inertial systems [3] and related disciplines [4,5,6]. Accelerometers, as the most basic inertial devices, are widely used in automobile, defense and impact measurement [7,8]. However, the requirements for accuracy are becoming higher and higher. Therefore, how to improve the accuracy of the accelerometer calibration process is very important. Many researchers have undertaken a lot of work to improve the accuracy of calibration. The approaches pursued include those described below.

Accelerometer denoising methods can be divided into the following two types: hardware-based methods and software methods. Zhang proposed a capacitive micro-electro-mechanical system (MEMS) accelerometer based on an asymmetrical anti-spring structure, which has high sensitivity and low noise performance. The accelerometer has been successfully applied in an earthquake [9]. Rao developed a micromachined micro-g capacitive accelerometer with a silicon-based spring-mass sensing element. By increasing the weight of the proof mass, the thermal noise was reduced [10]. Kamada devised a sensor architecture with a unique perforated and electrode-separated mass structure; the accelerometer achieved both low noise and low power consumption [11]. Yeh presented a monolithically integrated CMOS-MEMS three-axis capacitive accelerometer, which achieved the goal of low noise and low zero gravity [12]. Utz designed an accelerometer consisting of an ultra-low noise CMOS integrated readout-IC and a high-precision bulk micro-machined sensing element; the acceleration equivalent noise was significantly reduced [13]. Liu presented a low-power micromechanical capacitive accelerometer system with hybrid signal output to ensure a low noise output signal [14]. Najafi introduced classic and knowledge-based intelligent controllers for regulation of a vibratory MEMS accelerometer to filter stochastic noises [15]. Edalafar developed a high-performance micromachined capacitive accelerometer with the feature of low noise [16].

Another denoising method is that of software compensation. Algorithm compensation is more convenient and flexible than hardware-based compensation. Yan proposed a hybrid denoising algorithm based on time-frequency peak filtering (TFPF), local mean decomposition (LMD) and sample entropy (SE) to decrease the influence of noise on the high-g MEMS accelerometer (HGMA) output signal [17]. Guo presented a Kalman filtering method based on information fusion. By using the MEMS gyroscope and line accelerometer signals to implement the filtering function under the Kalman algorithm, the noise of the gyroscope signal was significantly reduced [18]. Zou combined ensemble empirical mode decomposition (EEMD) with time-domain integration. The fusion algorithm was based on a bridge dynamic displacement reconstruction method, then the high-frequency ambient noise was effectively eliminated [19]. Ding combined a wavelet de-noising method with a nonlinear independent component analysis (ICA) method to tackle the nonlinear BSS problem with additive noise [20]. Shen studied a novel multiple inputs/single output model based on a genetic algorithm (GA) and the Elman neural network (Elman NN) to improve the temperature drift modeling precision of gyroscopes [21]. Jiang adopted an artificial intelligence (AI) method to de-noise the MEMS inertial measurement unit (IMU) output signals [22]. Lu proposed a denoising method based on the combination of empirical mode decomposition (EMD) and wavelet threshold [23]. Zhu proposed a radial basis function (RBF) neural network (NN) + genetic algorithm (GA) + Kalman filter (KF) method, combined with a temperature drift model, so that noise characteristics were well optimized [24]. Zhang proposed a dual modulation method. The intensity modulation enabled movement of the signal to a high frequency, and the light source noise was suppressed perfectly by combining phase modulation [25]. Mokhtari implemented wavelet de-noising and de-trending techniques in order to filter angular accelerations [26]. He proposed the Kalman filter method to preprocess the raw data to reduce noise [27]. Abbasi-Kesbi presented a method based on the L2-norm total variation (LTV) algorithm. The obtained signals from the accelerometer were denoised [28]. Kou designed a hybrid algorithm based on forward linear prediction (FLP), a grey accumulated generating operation (AGO) and lifting wavelet transform (LWT) to achieve a better denoising effect [29]. Shen proposed a parallel processing algorithm, which was based on variational mode decomposition (VMD) and an augmented nonlinear differentiator (AND), to improve the effectiveness of the de-noising process [30]. Wang studied a method based on an improved ensemble local mean decomposition to reduce noise [31]. Cao proposed a structural equivalent electronic model and improved differential interface based on weak signal detection technology to improve the accuracy of inertial devices [32]. Wang used a method based on ensemble empirical mode decomposition (EEMD) and multipoint optimal minimum entropy deconvolution adjusted (MOMEDA) to reduce noise [33]. Cao introduced a sense mode closed-loop method for a MEMS gyroscope, which was based on the dipole temperature compensation method, and improved accuracy effectively [34]. Cai proposed a parallel processing model for eliminating gyroscope noise and temperature drift based on multi-objective particle swarm optimization based on a variational modal decomposition-time-frequency peak filter (MOVMD–TFPF) and the beetle antennae search algorithm– Elman neural network (BAS–Elman NN) [35]. Shen introduced a noise reduction algorithm based on an improved empirical mode decomposition (EMD) and forward linear prediction (FLP) to reduce the noise of a fiber optic gyroscope; the standard deviation of the gyroscope output signal was effectively reduced [36]. Cao proposed an IAWTD (improved adaptive wavelet threshold de-noising)-CSVM (C-means support vector machine)-EEMD (ensemble empirical mode decomposition) algorithm to compensate for the humidity drift of a gyroscope; this algorithm effectively reduced the quantization noise, bias stability and angle random walk of a MEMS gyroscope [37]. Ma introduced a fusion algorithm based on an immune-based particle swarm optimization (IPSO) improved VMD and BP neural network to reduce the temperature drift and output signal noise of the gyroscope [38]. Cao proposed a novel compensation method based on a permutation entropy local characteristic-scale decomposition (PE-LCD) and adaptive network-based fuzzy inference system (ANFIS) for a dual-mass MEMS gyroscope [39]. Ma introduced a parallel denoising model for a dual-mass MEMS gyroscope based on PE (Permutation entropy)-ITD (Intrinsic time scale decomposition) and SA (Simulated annealing)-ELM (Extreme learning machine) [40].

Although the above research institutions have been able to approximate the nonlinear relationship of the error using a variety of algorithm combinations, no significant suppression effect on the noise generated during the accelerometer calibration process has been demonstrated, and the characteristics of the original signal cannot be effectively preserved. To address the above problems, this paper proposes an accelerometer calibration process denoising algorithm based on empirical mode decomposition (EMD) and time-frequency peak filtering (TFPF). First, the intrinsic mode function (IMF) components of the high-frequency band after EMD decomposition are removed. Then, the TFPF algorithm is used to process the IMF components of the intermediate frequency, while the low-frequency IMF components are retained. Finally, the signal is reconstructed. The spectrum analysis results show that EMD + TFPF can effectively protect the characteristics of the original signal, and the error can be controlled within 0.5%. The results of Allan variance analysis show that the filtering effect is improved by 97.4% compared with the original data.

This paper introduces the proposed method as follows: Section 2 introduces the structure of the high g-value accelerometer, while Section 3 describes the construction of the EMD and TFPF fusion algorithm. The impact experiments performed on the accelerometer and signal processing are described in Section 4. Finally, Section 5 presents the conclusions of the paper.

## 2. Structure of MEMS Accelerometer and Working Mode Analysis

**Figure 1:**
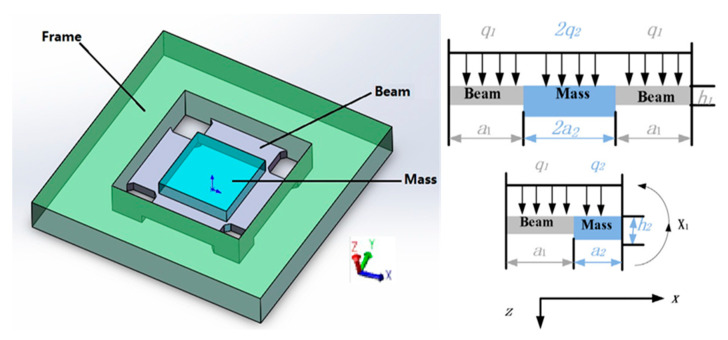
HGMA structure schematic and size.

The original signal collected in this paper is from a newly designed and manufactured high-g MEMS accelerometer (HGMA) [41,42]. It is a kind of accelerometer with a high impact survival rate and high measuring range. The detection method for the HGMA used is the piezo resistance and the output signal is the voltage. The HGMA adopts a four beam and island structure. The frame, four beams and the center mass are all rectangular, which is conducive to processing. The structure diagram and parameters are shown in Figure 1.

The coordinate system is constructed with the cross-section of the accelerometer. The central dividing line of the cross-section is the z-axis (specifying that the direction is positive to the downward direction). The other middle line is the x-axis, and the right direction is positive. The frame constructed is shown in Figure 1. The beam length, width and thickness are *a*_1_, *b*_1_ and *c*_1_, respectively; the mass length, width and thickness are 2*a*_2_, *b*_2_ and *c*_2_, respectively. The size values are shown in Table 1.

**Figure 2:**
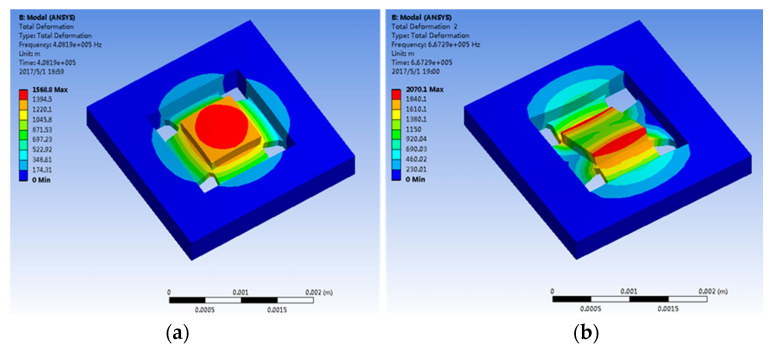

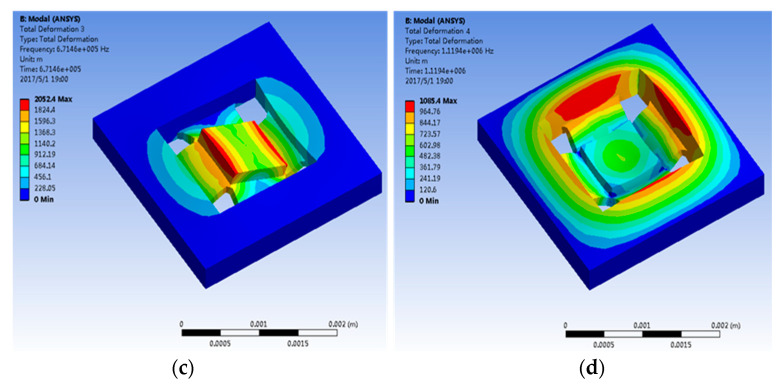
Mode simulation of HGMA structure. (**a**–**d**) are the 1st, 2nd, 3rd and 4th order modes.

The accelerometer is simulated and analyzed through ANSYS soft at four primary resonance modes; Figure 2a–d show the first, second, third and fourth modes, respectively. The first mode mass moves along the z-axis and is the working mode; the second mode mass rotates around the x-axis; the third mode mass rotates around the y-axis; the fourth mode mass and frame move along the z-axis. The resonant frequencies of the four modes are shown in Table 2, which indicates that the 1st order is the working mode of HGMA and its resonant frequency is 408 kHz. The 2nd-order mode resonant frequency is 667 kHz and has a 260 kHz gap with the 1st-order mode, which means that the coupling movement between these two modes is tiny and is good for HGMA linearity.

**Figure 3:**
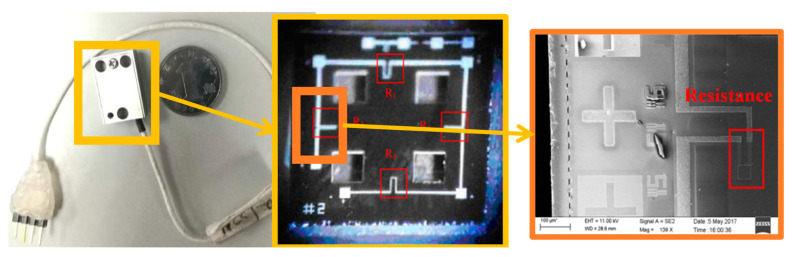
Overall photo, CCD photo and SEM photo of HGMA.

The structure of the HGMA is made of silicon bonding on glass. The main technological process is divided into 12 steps. SEM photos and CCD photos of the accelerometer structure are shown in Figure 3.

## 3. Methodology Combining EMD and TFPF

### 3.1. Empirical Mode Decomposition

Empirical mode decomposition (EMD) is an effective method for processing non-linear and non-stationary time-varying sequences. The method can adaptively decompose signals based on the time-scale characteristics of the data itself. The algorithm can decompose complicated time-series data into a finite number of intrinsic mode functions (IMF). The IMF component has all the fluctuation information of the original data at the corresponding time-scale. The specific steps of the EMD are as follows [43]:

Step 1: Find all the maximum points of the original signal *x*(t) and use the cubic spline interpolation function to form the upper envelope of the original data. Similarly, find all the minimum points and use the same interpolation function to form the lower envelope. The average of the upper and lower envelopes is set as *m*_1_; its value is shown in Equation (1).

(1) $$m_{1}=\frac{1}{2}\left(x_{\min}\left(t\right)+x_{\max}\left(t\right)\right)$$

where *x*_min_ and *x*_max_ represent the maximum envelope and the minimum envelope, respectively.

Step 2: Subtract the average value from the original signal to obtain a new high-frequency sequence with the low frequencies removed, denoted as *h*_1_(*t*).

(2) $$h_{1}\left(t\right)=x\left(t\right)-m_{1}$$

where *x*(*t*) is the original signal.

Step 3: If *h*_1_(*t*) is not an IMF component, set *h*_1_(*t*) as the original data, repeat the above process.

Continue with Step 1, obtain the average value *m*_11_.

(3) $$m_{11}=\frac{1}{2}\left(h_{1}{}_{\min}\left(t\right)+h_{1}{}_{\max}\left(t\right)\right)$$

Continue with Step 2, obtain *h*_11_(*t*).

(4) $$h_{11}\left(t\right)=h_{1}\left(t\right)-m_{11}$$

If *h*_11_(*t*) is still not an IMF component, repeat the loop *k* times and obtain *m*_1*k*_ and *h*_1*k*_(*t*) until *h*_1*k*_(*t*) meets the conditions of the IMF. The results are as follows:

(5) $$\;m_{1k}=\frac{1}{2}\left(h_{1}{{}_{k}}_{\min}\left(t\right)+h_{1}{{}_{k}}_{\max}\left(t\right)\right)$$

(6) $$h_{1k}\left(t\right)=h_{1k-1}\left(t\right)-m_{1}{}_{k}$$

Step 4: Assume *c*_1_(*t*) as the highest frequency component of the original signal, set the value as follows:

(7) $$c_{1}\left(t\right)=h_{1k}\left(t\right)\;$$

Step 5: Separate *c*_1_(*t*) from *x*(*t*) and obtain Equation (8).

(8) $$x_{1}\left(t\right)=x\left(t\right)-c_{1}\left(t\right)$$

where *x*_1_(*t*) is the new high-frequency sequence with low frequency removed.

Step 6: The value obtained by Equation (8) is subjected to the above sieving process, so the second IMF component can be obtained. After repeating *n* times, obtaining the *n*th IMF-compliant component, namely:

(9) $$\left\{\begin{array}{c} x_{1}\left(t\right)-c_{2}\left(t\right)=x_{2}\left(t\right)\\ \ldots \\ x_{n-1}\left(t\right)-c_{n}\left(t\right)=x_{n}\left(t\right) \end{array}\right.$$

Step 7: If the last component *x_n_* (*t*) is a monotonic function and its value is small or tends to zero, the decomposition is terminated and the steps are not repeated. Finally, the relationship between the original signal and each component is shown in Equation (10).

(10) $$x(t)=\sum_{i=1}^{n}c_{i}(t)+x_{n}(t)$$

where *c*_1_(*t*), *c*_2_(*t*), *c*_3_(*t*), …, *c_n_*(*t*) are the individual IMF components; *x_n_*(*t*) represents the average trend of the signal.

### 3.2. Time-Frequency Peak Filtering

For the noise filtering problem of low SNR (signal-to-noise ratio) signals, the time-frequency peak filtering (TFPF) filter works well. In this method, the noisy signal is coded and modulated into a certain analytical signal frequency, and the Wigner–Ville time-frequency distribution is used to obtain an estimate of the peak frequency of the analyzed signal. According to the characteristics of the modulation signal of the noise in the Wigner–Ville distribution, when the time-frequency peak is extracted, the influence can be filtered out; finally, the analytical signal is restored, and the signal noise reduction can be realized.

The time-frequency peak filtering method is very effective for the extraction of weak signals. It can be mainly divided into the following steps [44,45,46]:

Step 1: Assume that the noisy signal is as follows:

(11) $$s_{c}\left(t\right)=\;s\left(t\right)+c\left(t\right)$$

where *s_c_*(*t*) is the signal including noise, *s*(*t*) is the signal and *c*(*t*) is the Gaussian white noise.

Step 2: Encode the signal and obtain Formula (12).

(12) $$\begin{array}{l} z(t{)=e}^{j2\pi \mu \int_{-\infty }^{t}s_{c}(\lambda )d\lambda } \end{array}$$

where *z*(*t*) is the analytical signal and *μ* is the frequency modulation factor with a value range from 0–1.

The discrete expression of Formula (12) is as follows:

(13) $$\begin{array}{l} z(n{)=e}^{j2\pi \mu \sum_{i=0}^{n}s_{c}\left(iT_{s}\right)T_{s}} \end{array}$$

where *T_s_* is the sampling time.

According to the sampling theorem, the highest frequency of the sampling signal is *f_s_*/2 and the time domain discrete signal has no negative frequency. Then, set the instantaneous frequency of *z*(*n*) as follows:

(14) $$f_{i}(n)=\frac{ [\sum_{i=0}^{n}s_{c}(iT_{s})-\sum_{i=0}^{n-1}s_{c}(iT_{s})T_{s}]}{T_{s}}=s_{c}(iT_{s})$$

where *f_i_*(*n*) is the instantaneous frequency of *z*(*n*) with a frequency range between 0 and *f_s_*/2.

From the above analysis, Formula (15) can be obtained.

(15) $$0\leq s_{c}(iT_{s})\leq f_{s}/2$$

In general, for convenience of calculation, Formula (13) can be changed to the following:

(16) $$\begin{array}{l} z(n{)=e}^{j2\pi \mu \sum_{i=0}^{n}s_{c}\left(i\right)} \end{array}$$

From Equation (16), Equation (15) can be changed to the following:

(17) $$0\leq s_{c}(i)\leq 1/2$$

It can be derived from Formula (17) that the signal amplitude should be limited within the range and that there will be no overlap when it is modulated into a frequency. In order to avoid signal distortion during frequency modulation, scale the acquired signal [47]:

(18) $$s_{c}(i)=(a-b)\frac{s_{c}(i)-\min[s_{c}(i)]}{\max[s_{c}(i)]-\min[s_{c}(i)]}+b$$

where *b* ≤ *a*, and their values range from 0 to 0.5.

Step 3: Find the Wigner–Ville distribution (WVD) of the resolved signal [48].

(19) $$\begin{array}{l} W_{z_{x}}(t,f)=\int_{-\infty }^{\infty }z_{x}(t+\frac{\tau }{2})z_{x}{}^{*}(t-\frac{\tau }{2})e^{j2\pi f\tau }d\tau \end{array}$$

where *t* represents time, *τ* represents the integral variable, and *f* represents the frequency.

Since the actual signal has nonlinear characteristics, the pseudo-Wigner–Ville distribution (PWVD) is used to find the time-frequency distribution of the signal to ensure that the estimation of the signal in the window is unbiased [46].

The PWVD distribution of the noisy signals is as follows:

(20) $$W_{pz_{x}}(t,f)=\int_{-\infty }^{\infty }h(\tau )z_{x}(t+\frac{\tau }{2})z_{x}{}^{*}(t-\frac{\tau }{2})e^{j2\pi f\tau }d\tau$$

where *h*(*τ*) is a real-valued window function.

The discrete expression of Formula (20) is as follows:

(21) $$W_{pz_{x}}(n,m)=\sum_{l=-L}^{L}h(l)z_{x}(n+l)z_{x}{}^{*}(n-l)e^{j2\pi mn}$$

where *h*(*l*) is a window function, and its width is 2L + 1.

The maximum value of the time-frequency distribution of the analytical signal is taken as the estimate of the instantaneous frequency by the frequency variable, which is shown in Formula (22).

(22) $${\hat{f}}_{z}=\frac{1}{\mu }\arg_{f}\max [W_{pz_{x}}(t,f)]$$

Step 4: Signal reduction

The instantaneous frequency is the estimate of the effective signal of the original signal. Set the estimate of the effective signal of the original signal as follows:

(23) $${\hat{S}}^{\prime }(t)={\hat{f}}_{z}$$

Perform an inverse scaling of the estimate of the effective signal and obtain the Formula (24) [47]:

(24) $$\hat{S}(i)=\frac{\left({\hat{S}}^{\prime }(i)-b)(\max(s_{c}(i))-\min s_{c}[(i)]\right)}{\left(a-b\right)}+\min[s_{c}(i)]$$

If, when filtering, the effect (such as the signal-to-noise ratio) is not ideal, step 1 can be returned to for iterative filtering.

### 3.3. The EMD-TFPF Fusion Algorithm

The TFPF method can suppress part of the random noise. However, the TFPF method may not have a very good effect. However, the EMD algorithm can also filter but can lead to serious signal distortion. This section proposes a fusion algorithm based on empirical mode decomposition (EMD) and time-frequency peak filtering (TFPF).

**Figure 4:**
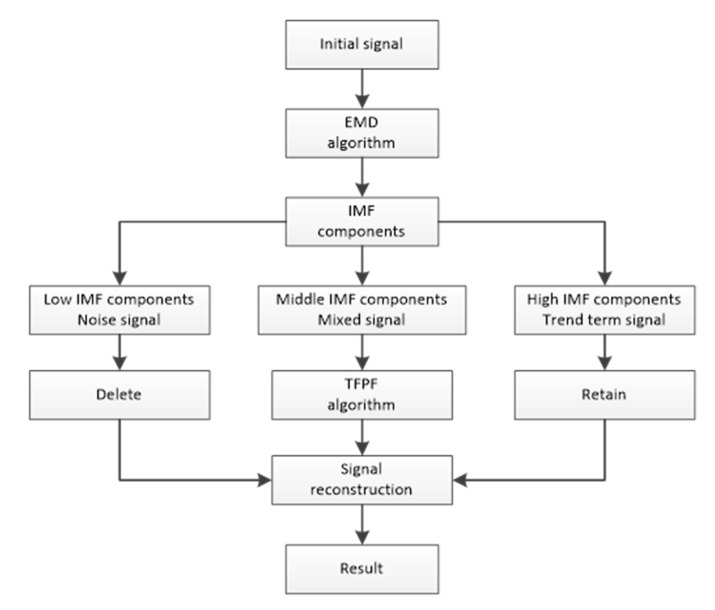
The process of the EMD-TFPF fusion algorithm.

Firstly, the EMD algorithm is used to decompose the acceleration calibration signal. The processed signal is divided into many IMF components, which are distributed from high frequency to low frequency. The decomposed IMF component is mainly divided into three parts: a noise part, a mixed signal and a trend term. There is almost no useful signal component in the high-frequency signal, so it can be ignored, which can effectively suppress part of the random noise. Then, we select a small amount of IMF for signal reconstruction. The TFPF algorithm is then implemented for each selected IMF to further filter the random signals generated in the calibration process; the specific approach uses a small window PWVD to suppress the residual weak random noise in the time-frequency analysis. Finally, the required signals are restored by adding the processed IMFs. The specific process is shown in Figure 4.

The selection of IMF components is an important problem. In the calibration process of the accelerometer, there are two frequencies that need to be considered. The first is the shock frequency in the calibration process, and the second is the vibration frequency of the accelerometer. For the selected IMF components, spectrum analysis of each IMF component shows that there will be peaks around the shock frequency and vibration frequency. In order to ensure that the calibration process of the MEMS accelerometer does not produce spectrum distortion, the spectrum characteristics of the original signal should be retained as far as possible. Therefore, for the selection of these IMF components, the IMF component whose spectral energy ratio is greater than the average spectral energy of the IMF component is selected here.

EMD-TFPF is an improvement of the two filtering algorithms EMD and TFPF. The EMD-TFPF algorithm can effectively separate the signal noise and process each part of the IMF component accordingly. It avoids the problem that the real signal is obtained by EMD decomposition but the noise cannot be effectively filtered. It also avoids the problem that the window selection in TFPF is too large and affects the characteristics of the original signal.

## 4. Experiment and Signal Processing

**Figure 5:**
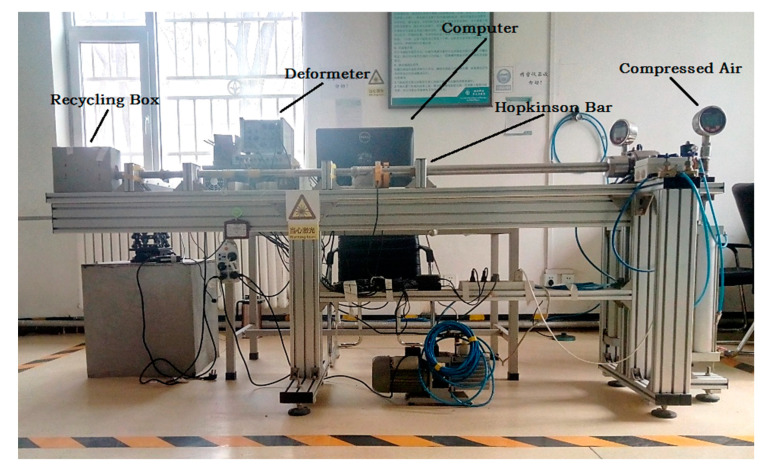
Hopkinson bar calibration system.

As shown in Figure 5, the Hopkinson bar calibration system included a recycling box, deformeter, computer, Hopkinson bar, and compressed air. The power supply provided a +5 V voltage to the HGMA, the temperature was maintained at 25 °C (room temperature value), and the sampling rate was set to 20 MHz.

**Figure 6:**
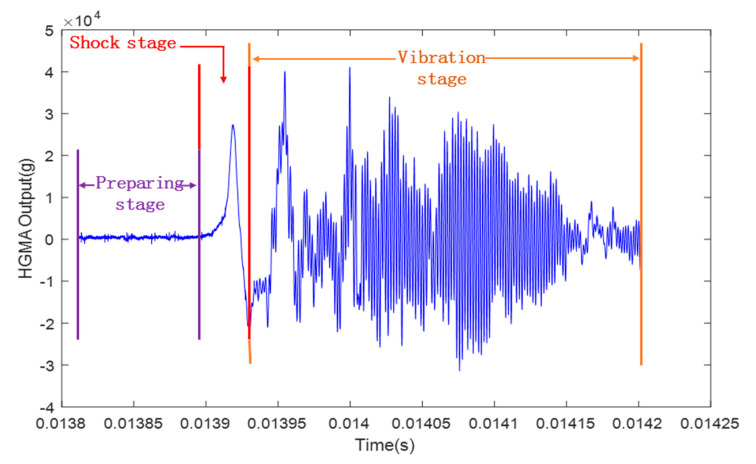
The output of the MEMS accelerometer in the calibration experiment.

HGMA was calibrated in the Hopkinson bar calibration system. The HGMA output signal was acquired using a high-speed data acquisition system and a computer. The results are shown in Figure 6.

It is worth noting that the HGMA calibration process is mainly divided into three phases, namely the preparation phase, the impact phase and the oscillation phase.

1. Preparation stage: this part mainly includes the bias characteristics of the HGMA. The noise signal is included. The maximum peak noise is approximately 1000 g.
2. Shock stage: this stage is the main part of the accelerometer calibration experiment; the output peak value is about 28,030 g and the pulse width is about 10 μs.
3. Vibration stage: this part mainly captures the vibration information for the HGMA, reflecting the dynamic characteristics of the HGMA.

**Figure 7:**
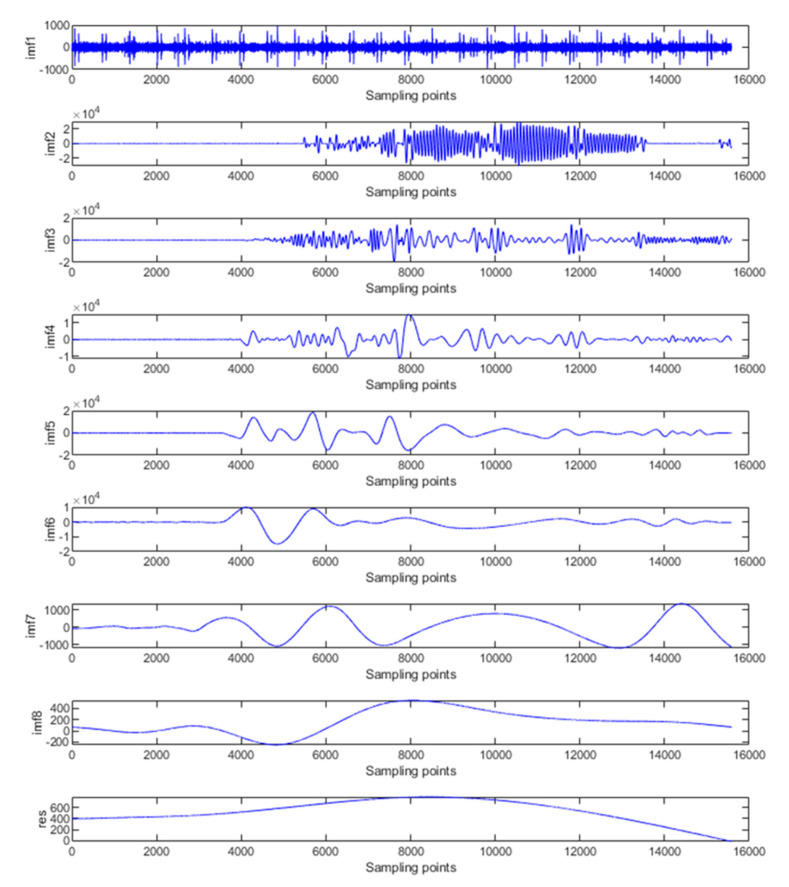
EMD decomposition of original signal.

The accelerometer calibration signal is decomposed by the EMD algorithm, as shown in Figure 7. It can be seen that the acceleration signal is finally decomposed into eight IMF components and a residual component. The IMF components are distributed from high frequency to low frequency.

According to the EMD low-pass filtering de-noising processing algorithm, the high-frequency part of the IMF component is removed directly, and the medium- and low-frequency IMF components are processed by Section 3.3. Therefore, each IMF component is finally layered. IMF1 is the noise term, while IMF2, IMF3, and IMF4 are mixed items, and IMF5–IMF8 are the trend items of the signal and should be reserved.

**Figure 8:**
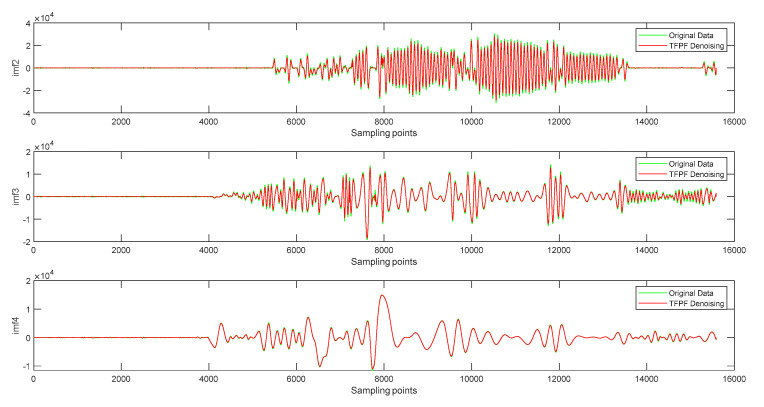
The results of mixed components after TFPF de-noising.

The mixed terms are processed by the TFPF algorithm, as shown in Figure 8. By observing the time series, it can be found that the TFPF algorithm can effectively suppress the random noise based on protection of the useful signal.

After the above signal processing, the accelerometer signal is reconstructed according to the steps in Figure 4. The results of reconstructing the accelerometer signal using the three methods are shown in Figure 8. It can be seen from Figure 8 that EMD has the best effect in the three stages.

In the preparation stage, the maximum noise of the original signal, the EMD denoising signal, the TFPF denoising signal, and the EMD + TFPF denoising signal were 1958 g, 878.7 g, 1483 g, 811.6 g, respectively. According to the results, among the three methods, the TFPF method had the worst denoising effect, and the EMD + TFPF method had the most obvious denoising effect.

In the shock stage, the curves of the original signal, the EMD denoising signal, the TFPF denoising signal, and the EMD + TFPF denoising signal almost coincided. The results show that the three algorithms contained the same information as the original signal.

In the vibration stage, the TFPF and EMD + TFPF methods captured the original signal well, but the EMD method appeared to result in distortion and did not fully reflect the original data information.

In summary, The EMD method was able to effectively suppress the random signal of the calibration process but failed to effectively protect the original signal characteristics of the accelerometer. The TFPF algorithm could also filter out a lot of the random signals, but the effect was less obvious. Only by combining the EMD and TFPF algorithms was the random signal effectively suppressed and the basic characteristics of the reserved signal ensured.

**Figure 9:**
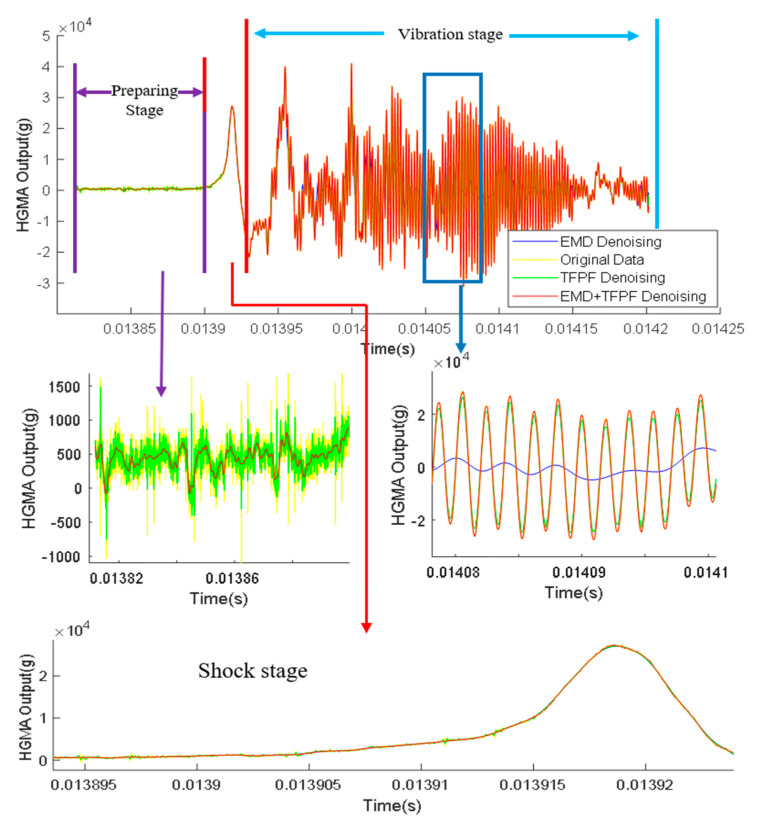
Noise reduction results at different stages.

For the spectrum analysis of the accelerometer, the main focus was on the three states of the accelerometer calibration process. The first stage is the preparation stage, the second stage is the shock stage, and the third stage is the resonant frequency of the vibration stage (Figure 9 shows the three stages).

**Figure 10:**
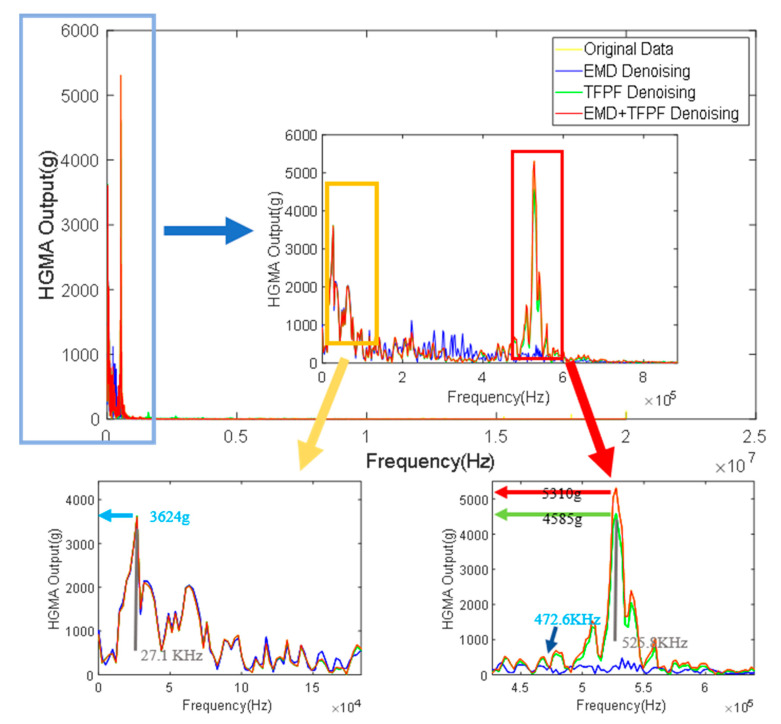
Frequency characteristics comparison of different denoising results.

The spectrum analysis results of the signals processed by the different algorithms are shown in Figure 10. For the shock stage: the frequency peak was approximately 27.1 kHz. During this stage, the amplitudes of the original signal data, the EMD, TFPF and EMD + TFPF denoising signals were 3608 g, 3541 g, 3624 g and 3610 g, respectively. Therefore, the true amplitude and frequency information of the original signal was able to be captured by the three denoising methods.

For the vibration phase: the frequency peak was approximately 525.8 kHz and the amplitudes of the original signal data, the EMD denoising result, the TFPF denoising result, and the EMD + TFPF denoising result were 5284 g, 472.6 g, 4585 g, 5310 g, respectively. The amplitude and shape of the original signal data, and the TFPF and the EMD + TFPF denoising results, were basically the same. The EMD denoising method distortion prevented accurate reflection of the frequency and amplitude information of the original data.

**Figure 11:**
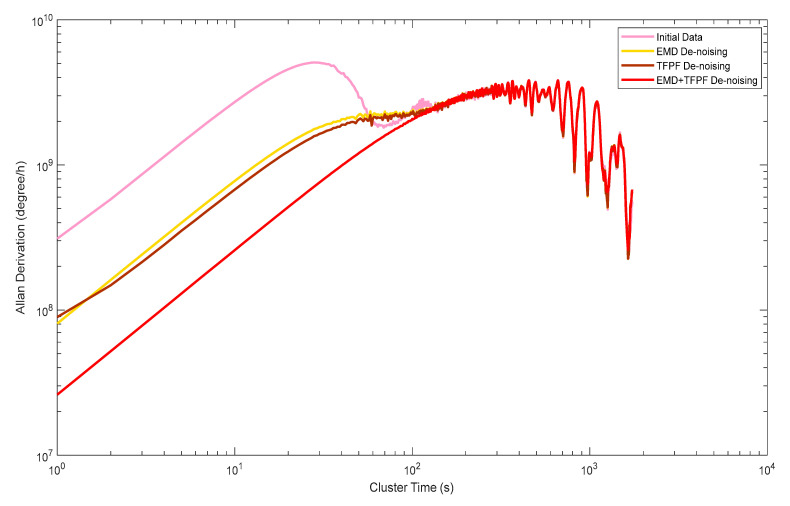
Allan analysis results of the denoising method during the “preparation stage”.

The results of the several denoising methods in the preparation stage were evaluated using Allan analysis of variance, as shown in Figure 11. It can be seen that the random walking and bias stability of the original data was 6.9202 × 10^8^ (g/h), which improved to 8.102 × 10^7^ (g/h), 8.8901 × 10^7^ (g/h), and 1.8002 × 10^7^ (g/h) using the EMD method, the TFPF method and the EMD + TFPF method, respectively. The results indicate that the effect of the fusion algorithm was the most obvious among the three methods.

**Figure 12:**
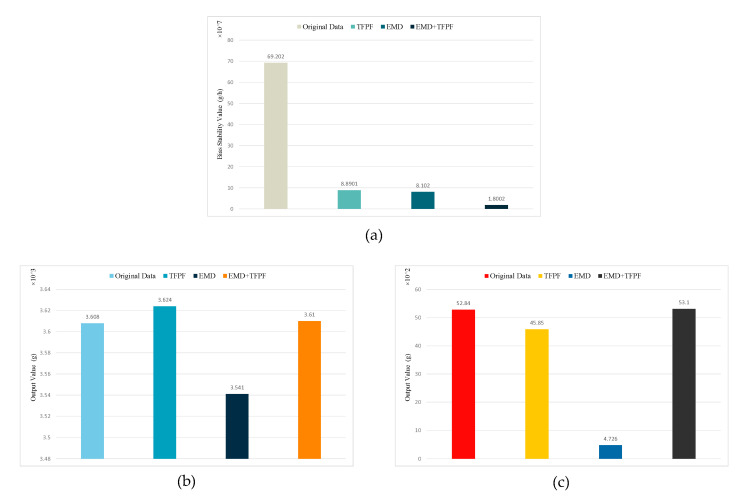
Noise reduction effect of the three algorithms at different stages. (**a**) is the increase in the bias stability value in the preparation stage, (**b**,**c**) are the optimization results in the shock stage and the vibration stage, respectively.

The denoising results of the different methods at different stages are shown in Figure 12. Compared with the EMD method and the TFPF method, the EMD + TFPF method worked best in the three stages.

It was calculated that, in the preparation stage, the noise of the original signal was reduced by 88.3%, 87.2% and 97.4% using the EMD, TFPF, and EMD + TFPF denoising methods, respectively. In the shock stage, the errors from the original three denoising methods of EMD, TFPF, EMD + TFPF were 0.94%, 0.44%, and 0.055%, respectively. In the vibration stage, the errors for the EMD, TFPF, and EMD + TFPF methods were 91.06%, 13.23%, and 0.49%, respectively.

The results for the preparation stage showed that the EMD + TFPF method achieved the best denoising effect. In the shock stage and the vibration stage, the EMD + TFPF method did not destroy the original calibration data, and was able to perform data processing during the calibration process.

## 5. Conclusions

This paper introduced the high-g accelerometer and the filtering methods used in the accelerometer calibration process. The structure of the accelerometer was analyzed using finite element analysis software. Simulation results showed that the resonant frequency of HGMA in the 1st-order mode was 408 kHz. The 2nd-order resonant frequency was 667 kHz and the gap with the 1st-order mode was 260 kHz, so the coupling movement between the two modes was small, and the HGMA had better linearity. For noise reduction of the accelerometer calibration, the EMD + TFPF method was proposed. The method was mainly divided into the following steps: First, the IMF component of the high frequency band after EMD decomposition was removed; second, the IFPF algorithm was used to process the IMF component of the middle frequency band; third, the IMF component of the low frequency band was reserved; finally, the signal was reconstructed. The experimental results showed that the EMD + TFPF method was able to not only achieve a significant filtering effect, but also to retain the original signal to a large extent.

## Figures and Tables

**Table 1:** Structural parameters of the HGMA.

	Beam			Mass		
Parameters	length (*a*_1_)	width (*b*_1_)	height (*c*_1_)	length (*a*_2_)	width (*b*_2_)	height (*c*_1_)
size/μm	350	800	80	800	800	200

**Table 2:** Resonant frequencies of the four modes.

Mode Shapes	1	2	3	4
Resonant Frequency /kHz	408	667	671	1119

## Data Availability

The data used to support the findings of this study are available from the corresponding author upon request.
